# Effect of Thermally Reduced Graphene on the Characteristics and Performance of Polysulfone Mixed Matrix Ultrafiltration Membranes

**DOI:** 10.3390/membranes13080747

**Published:** 2023-08-21

**Authors:** Omnya Abdala, Ahmed Nabeeh, Abdul Rehman, Ahmed Abdel-Wahab, Mohammad K. Hassan, Ahmed Abdala

**Affiliations:** 1 Chemical Engineering Program, Texas A&M University at Qatar, Doha P.O. Box 23874, Qatar or o.abdalla@gord.qa (O.A.); ahmed.mohamed1@qatar.tamu.edu (A.N.);; 2 Gulf Organization for Research & Development (GORD), Qatar Science & Technology Park, Tech1 Bldg, Suite 203, Doha P.O. Box 210162, Qatar; 3 Center for Advanced Materials (CAM), Qatar University, Doha P.O. Box 2713, Qatar

**Keywords:** Polysulfone membranes, mixed matrix membranes, thermally reduced graphene, oil–water separation, air-dehumidification

## Abstract

Ultrafiltration (UF) polymeric membranes are widely used in water treatment and support desalination and gas separation membranes. In this article, we enhance the performance of Polysulfone (PSF) mixed matrix membranes (MMMs) by dispersing different concentrations of thermally reduced graphene (TRG) nanofillers. The UF PSF-TRG MMMs were fabricated via the phase inversion process, and the impact of TRG loading on the characteristics of the membrane, including hydrophilicity, porosity, roughness, and morphology, were analyzed using a contact angle measurement, atomic force microscopy (AFM), scanning electron microscopy (SEM), and dynamic mechanical analysis. Incorporating TRG into the PSF matrix led to favorable effects in the instantaneous de-mixing during phase inversion, increasing the porosity and hydrophilicity of MMMs and improving the mechanical properties of the membranes. Moreover, membrane performance was examined to remove dispersed oil from oil–water emulsion and support air-dehumidification membranes. MMM performance in terms of flux and oil rejection was superior to the control PSF membrane. Incorporating 0.25% TRG into PSF resulted in a 70% water flux increase and higher oil rejection compared to the control PSF membrane. As a support for air-dehumidification membranes, the MMM also demonstrated enhanced humidity reduction and an over 20% increase in water vapor permeance over the control PSF membrane. These results indicate that the PSF-TRG MMMs are an excellent candidate for reliable oil–water separation and as a support for air-dehumidification membranes.

## 1. Introduction

Membrane technology offers numerous advantages over conventional techniques, including low energy consumption, reduced environmental impact, small footprint, and high reliability [1,2]. In the field of water treatment and gas separation, the use of polymeric membranes has experienced significant growth. Specifically, PSF has emerged as an exceptional material for the fabrication of low-pressure membranes, such as microfiltration (MF) and ultrafiltration (UF) membranes, which find application in water treatment and pretreatment for reverse osmosis desalination.

Oily wastewater containing organic pollutants is a significant environmental concern [3,4,5]. Conventional treatment methods such as flotation, coagulation, and biological treatment often fall short due to the dispersion and small size of oil droplets [6,7,8]. Advanced membrane technology, particularly, UF membranes, offers an effective solution, especially for oil droplets <20 μm, with easy operation, low costs, high separation efficiency, and no by-products [9,10]. However, challenges persist with polymeric UF membranes such as fouling, aging, and a tradeoff between selectivity and permeability [1,2]. In parallel, air cooling based on membrane air-dehumidification has emerged as an alternative to conventional mechanical vapor-compression cooling, offering benefits in energy savings, environmental sustainability, and economic feasibility [11,12,13,14,15]. This approach involves hydrophilic membranes for isothermal water vapor separation from ambient air, allowing for efficient dehumidification and a comfortable humidity level [16,17,18]. Among various options, thin film composite membranes supported on porous UF membranes like PS are excellent candidates due to their flexibility, ease of fabrication, and high water/air selectivity. However, their water vapor permeance (WVP) is currently limited by the WVP of the porous support, necessitating modification for commercial application [19,20,21,22].

There is growing interest in the use of carbon-based nanofillers in polymeric membranes due to their excellent structural orientation, physical properties (including strong mechanical and electrical properties), high aspect ratio, chemical stability, and economic favorability [23,24]. These nanomaterials encompass carbon nanotubes, graphene, graphene oxide, and reduced graphene [25].

Recent studies have demonstrated the potential of incorporating nanofillers to enhance polymeric membrane performance for oil/water separation. Specifically, Lai et al. reported a 31.73% increase in water flux for polyethersulfone (PES) MMMs with a 0.75/0.25 weight ratio of hydrous manganese oxide (HMO) to titanium dioxide (TiO_2_) nanofillers, reaching a water flux of 28.4 L/m^2^·h without sacrificing the >97% oil rejection compared to the neat PES membrane [26]. Importantly, they also achieved a 91.5% flux recovery after fouling, which was attributed to the hydrophilicity imparted by the HMO. Similarly, Ang et al. showed a 32% increase in pure water flux from 398 to 524 L/m^2^·h and an 85% flux recovery ratio using 0.2 wt.% organo-modified montmorillonite (O-MMT) in cellulose acetate MMMs. The expanded interlayer spacing of O-MMT compared to pristine montmorillonite improved membrane porosity and hydrophilicity [27].

However, there remain challenges, such as membrane fouling, which can drastically reduce flux. For instance, Pagliero et al. reported a 50% flux reduction in polyvinylidene fluoride (PVDF) membranes during oil degumming, attributed to wax fouling interactions [28]. To combat this, Abdalla et al. incorporated 0.2 wt.% aspartic acid-functionalized graphene oxide into PSF MMMs, resulting in a 97% increase in permeability to 1375 L/m^2^·h, achieving 97.9% oil rejection and 90% flux recovery after fouling cycles. They demonstrated that incorporating functionalized graphene oxide significantly improved the membrane’s hydrophilicity, porosity, and mechanical properties by about 13 degrees, 30.8%, and 74%, respectively. However, they faced challenges such as weak nanoparticle adhesion and difficulties in scaling up [29].

The incorporation of carbon-based nanofillers, specifically, has shown remarkable advancements. For instance, Ong et al. reported a 63% increase in water flux for PVDF membranes modified with 0.075 wt.% polydopamine (PDA) coating, reaching 52 L/m^2^·h. The PDA coating also enhanced the oil rejection rate from 92.3% to 98.8% for PVDF while maintaining 91.3 LMH/bar permeability [30]. Despite these improvements, the modifications often require specialized equipment and toxic reagents.

Graphene-based materials have shown exceptional potential in this field. For example, Peng et al. fabricated reduced graphene oxide and silica nanohybrid membranes which demonstrated high oil rejection and dye removal capabilities [31]. Similarly, Chen and Chen developed graphene oxide-coated stainless steel meshes that efficiently separated oil/water mixtures with 99.49% efficiency while exhibiting good fouling resistance. However, these methods were faced with issues related to the surface deposition technique and stability under high temperature conditions [32]. Furthering this, Zhao et al. produced impressive results with a polygorskite/graphene oxide MMM, achieving a water flux of 3734 L/m^2^·h·bar and >99.9% oil rejection [33].

Moreover, Qian et al. showed that TiO_2_/graphene oxide/silver nanoparticle membranes separated oil/water emulsions with 99.6% efficiency while degrading solubilized contaminants [34]. Yet, the use of ultraviolet irradiation, the stability of the dopamine linkage, and the low mechanical strength of some membranes highlighted the need for further research to overcome these challenges. Despite these advancements, the complexities of incorporating nanofillers, including functionalization, distribution, and adhesion, call for further exploration and innovation in this field.

Here, we developed a proficient technique to prepare TRG and incorporate prepared TRG into a PSF matrix. The effect of TRG loading in the PSF-TRG nanocomposite on membrane morphology, hydrophilicity, and mechanical properties is investigated. The performance of the fabricated MMMs for removing hydrocarbon oil from oil–water emulsion is examined. Lastly, the control and optimum MMM performance as a support for air-dehumidification membranes were analyzed by measuring their WVP and water/air selectivity.

## 2. Materials and Methods

### 2.1. Materials

Commercial graphite oxide (GO: SE2430) was purchased from Sixth Element Materials Technology Co., Ltd., Changzhou, China. Dimethylacetamide (DMAc), Polysulfone (PSF: Mn~22,000), and polyvinylpyrrolidone (PVP: Mw~55,000) were obtained from Sigma Aldrich, St. Louis, MO, USA, and directly used without further purification. A diesel sample obtained from Waqood petrol station (Doha, Qatar) was used for the oil–water emulsion.

### 2.2. TRG Synthesis and Characterization

**Figure 1:**
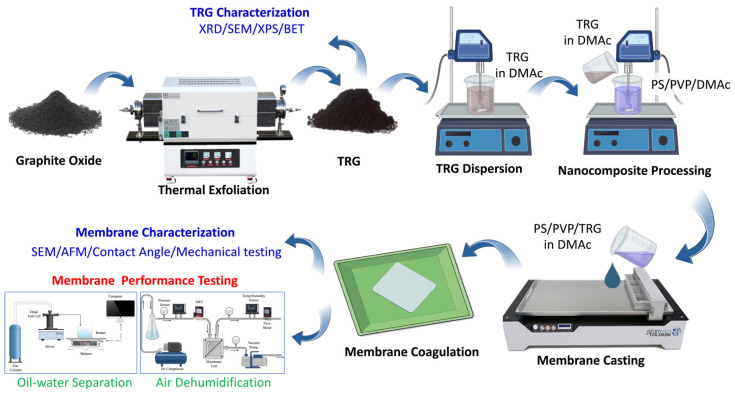
Flowchart of TRG preparation and characterization of PSF-TRG MMM membrane fabrication, characterization, and testing.

TRG was synthesized via thermal exfoliation of commercial Hummer GO [35], as shown in Figure 1. In brief, GO was dried at 60 °C for 24 h. Then, 1 g of GO was placed inside a 1″ closed-end quartz tube and purged with N_2_ gas at 100 mL/min for 10 min. The quartz tube containing the sample was inserted in a tube furnace equilibrated at 1000 °C and maintained for 30 s. Finally, the TRG sample was cooled to room temperature. The TRG’s morphology and crystal structure were analyzed using SEM (FEI Quanta 400 SEM, Thermo Fisher Scientific, Waltham, MA, USA). X-ray diffractometer (XRD: Ultima IV, RIGAKU, The Woodlands, TX, USA) with Cu-α radiation (λ = 0.15418 nm) operating at 40 kV and 20 mA was used to determine the stacking characteristics and interlayer spacing of TRG using Bragg’s law [30]. The surface area, porosity, and pore size distribution were measured via Nitrogen Adsorption using ASAB 2020 (Micromeritics, Norcross, GA, USA).

### 2.3. Membrane Fabrication

UF TRG membranes were fabricated using the phase inversion method [24] as shown in Figure 1. The control polymer solution was prepared by mixing 85% DMAc, 3% PVP (a pore-forming agent), and 17% PSF. The effect of the TRG filler in the membranes was studied by varying the concentration of TRG between 0% and 1% (0%, 0.1%, 0.25%, 0.5%, and 1%). To ensure the dope solutions’ homogeneity, the sample was bath-sonicated for 1 h, followed by probe sonication for 5 min and degassing for 5 min. Afterward, the dope solution was cast using a membrane-casting machine on a glass plate with a 150 μm casting knife. The cast films were undisturbed for 30 s under ambient conditions before being immersed in a deionized water coagulation bath for 5 min. Finally, for complete phase inversion, the membrane sheets were stored in deionized water that was changed daily for five days before further characterization and testing.

### 2.4. Membrane Characterization

The shape and structure of the prepared TRG membranes were studied through scanning electron microscopy (SEM) (FEI Quanta 400 SEM, Thermo Fisher Sci.), where explicit images of the surface topography were obtained. The hydrophilicity of the prepared membranes was analyzed by measuring the contact angle via the Kruse drop shape analyzer (KDSA: DSA25, Hamburg, Germany) through the sessile drop technique. Atomic force microscopy (AFM: Dimension Icon, Bruker, Billerica, MA, USA) was used to understand the effect of TRG loading on the roughness of the fabricated membranes, through which 10 × 10 µm and 3 × 3 µm three-dimensional images were taken under tapping mode. A dynamic mechanical analyzer (DMA: Q800, TA Instruments, New Castle, DE, USA) was utilized to investigate the fabricated membranes’ mechanical properties, i.e., Young’s modulus, strength, and ductility.

### 2.5. Oil Separation Membrane Performance Testing

The permeability of the prepared membranes was tested by measuring the pure water flux across the membrane using a dead-end cell (Sterlitech HP4750, Kent, WA, USA. Various trans-pressures were applied in the test. Thus, the pure water fluxes were calculated using the following equation:

(1) $$J=\frac{m}{t\times A}$$

where *J* is the pure water flux (L/m^2^·h), *m* is the volume of water permeated (L), *t* is the operating time (h), and *A* is the active membrane area ($14.6\times 10^{-4}\;m^{2}).$

The oil–water separation efficiency of the membranes was evaluated by calculating the percentage of oil rejection. The concentration of the oil emulsion in the feed was 100 ppm. Three oil permeate samples were collected from the dead-end cell. The carbon content of feed and permeate samples was analyzed using a total organic carbon analyzer (TOC: TOC-L, Shimadzu, Kyoto, Japan). The percentage of oil rejection was calculated as follows:

(2) $$R\left(\%\right)=\frac{C_{f}-C_{p}}{C_{f}}\times 100$$

where *C_p_* and *C_f_* are the TOC concentrations of oil permeate and feed, respectively.

### 2.6. Water Vapor Permeation Performance Analysis

**Figure 2:**
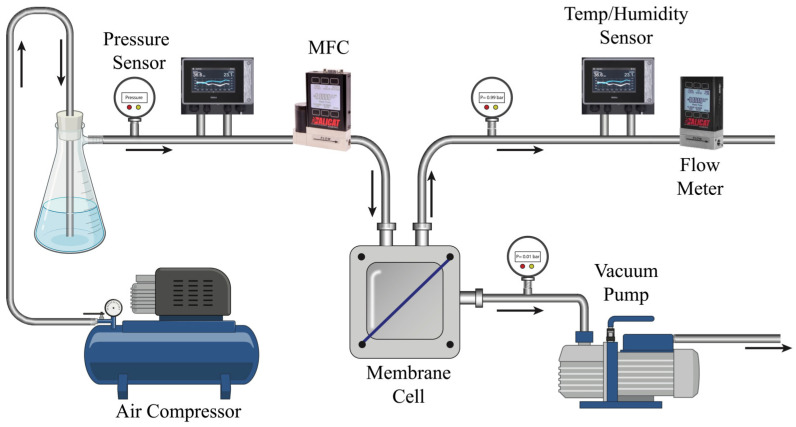
Testing setup for air-dehumidification membrane performance. Arrows represent the direction of flow.

The air-dehumidification performance of the membrane was tested at specific relative humidity levels and selected inlet feed pressures. The setup comprised an air-dehumidification module with a humidity controller, following the process developed by Culp [36]. Humidified inlet air entered the membrane cell, which released dry air and allowed water vapor to pass through the permeate side, facilitated by a vacuum pump, as shown in Figure 2.

The membrane’s performance as a support for air-dehumidification membranes was evaluated by measuring WVP. The experimental setup consisted of an air humidifier and a vacuum membrane cell, as illustrated in Figure 2. Compressed air was humidified to achieve a relative humidity of 90% and directed to the vacuum membrane module. The permeate side of the membrane cell was maintained at 2 mbar using an IKA Vacstar pump.

A mass flow controller (Alicat Scientific MFC5, New Market, MD, USA) was employed to control the inlet airflow, ensuring a constant flow rate of 2 standard liters per minute (SLPM) with an accuracy of 0.5%. The flow rate of the retentate stream was measured using a mass flow meter (Alicat Scientific MF5). The temperature and humidity of both the feed and retentate streams were measured using Vaisala Indigo500 humidity transmitters with HMP probes, providing measurements with an accuracy of 1.5%. The pressure within the feed, retentate, and permeate streams was continuously monitored using TC-direct 716–184 industrial pressure transmitters. These transmitters are equipped with a pressure range of 0 to 1.5 bar and maintain an accuracy level of 0.5%.

The membrane permeance was calculated using the following formula:

(3) $$\bar{P_{w}}=\frac{N_{w}}{A_{m}(\frac{P_{F_{w}}+P_{R_{w}}}{2}-{P_{P}}_{w})\;}$$

where $\bar{P_{w}}$ is the water permeance in (mol/m^2^ s Pa), $N_{w}$ is the water vapor flow rate across the membrane (mol/s), and $A_{m}$ is the active membrane area (15 × 10^−4^ m^2^). ${P_{F}}_{w}$, ${P_{R}}_{w}$, ${P_{P}}_{w}$ represent the partial pressure of the water at the feed, retentate, and permeate side respectively.

## 3. Results and Discussions

### 3.1. TRG Characterization

TRG was prepared following the thermal exfoliation/reduction of GO at a high temperature. In this process, the thermal reduction involves the removal of oxygen functional groups (-OH and C-O-C) on the GO surface as gaseous products. The thermal exfoliation/reduction of GO is a suitable process for the large-scale production of reduced graphene [37].

**Figure 3:**
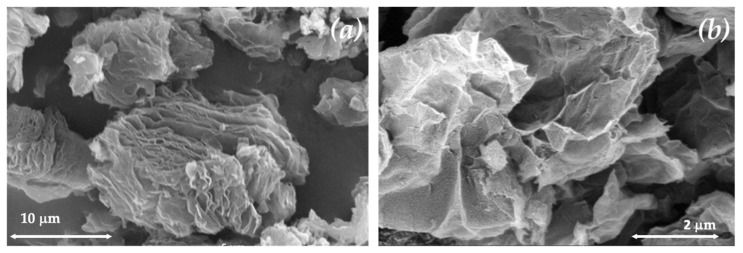

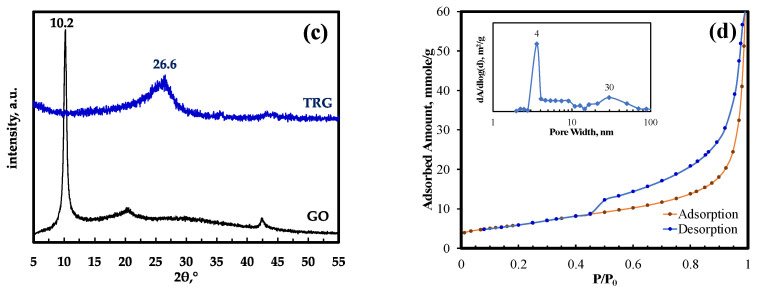
Surface morphology and characterization of TRG: (**a**) SEM Image: Illustrating the surface morphology of TRG. (**b**) SEM image: providing further surface morphology characterization of TRG at higher magnification. (**c**) XRD pattern showing the characteristic comparison of GO and TRG. (**d**) Brunauer–Emmett–Teller (BET) plots of N_2_ adsorption–desorption isotherms at 77 K and Barrett–Joyner–Halenda (BJH) pore–width distribution analysis for TRG. The inset in the top-left corner shows the BJH plots of the differential surface area versus pore width for TRG.

The surface morphology of the TRG was examined using SEM and is shown in Figure 3a,b. The TRG morphology reveals a combination of thin graphene sheets that form a nonporous wrinkled structure. The crystal structure of TRG was examined using XRD, and its corresponding pattern and the pattern for the starting GO are provided in Figure 3c. The oxidation of GO was evident upon increasing the d-spacing to 8.7 Å (2θ = 10.2°). This peak in the GO pattern is absent from the TRG pattern, indicating the exfoliation of GO to TRG. The broad peak in the TRG pattern at 2θ = 26.6° suggests the existence of a fraction of not fully exfoliated structures. The surface area, pore volume, and pore size are important factors that affect the performance of adsorbents. Therefore, the adsorption/desorption of N_2_ at 80 K was used to analyze these parameters. Figure 3d shows the adsorption/desorption isotherm for the TRG. The N_2_ adsorption isotherm for TRG shows the presence of a hysteresis loop associated with capillary condensation in mesopores that is typical of an IUPAC type IV isotherm. The pore volume was determined by applying the Barrett–Joyner–Halenda analysis for lower pressures. The Kelvin equation was used to determine the pore size distribution of TRG. The surface area of TRG is 2.48 m^2^/g, and its pore volume is 2.09 cm^3^/g, corresponding to a porosity of 82%. This high TRG porosity is consistent with the structure probed using SEM.

### 3.2. Membrane Characterizations

**Figure 4:**
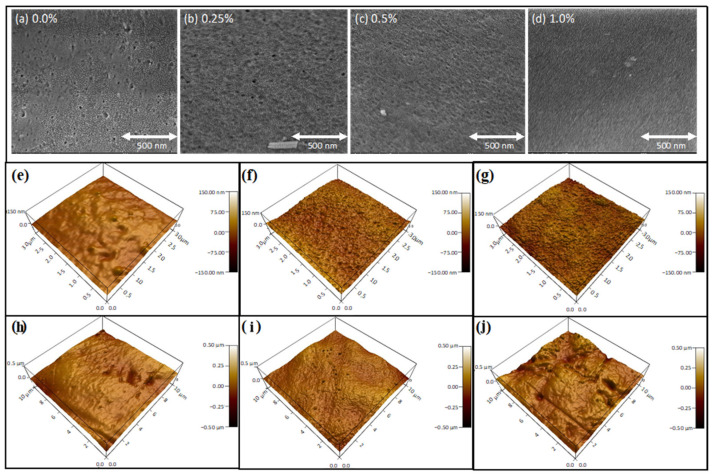
Surface topography analysis of TRG membranes: (**a**–**d**) SEM and optical images: illustrating the surface morphology of PSF-TRG MMMs with different TRG concentrations: (**a**) 0%, (**b**) 0.1%, (**c**) 0.25%, (**d**) 0.5 wt.% (**e**–**g**). Three-dimensional AFM images with a scan size of 3 × 3 µm, showcasing the surface topography at varying TRG concentrations: (**e**) 0%, (**f**) 0.25%, (**g**) 1%. (**h**–**j**) AFM Images with a Scan Size of 10 × 10 µm, highlighting the surface topography at different TRG concentrations: (**h**) 0%, (**i**) 0.25%, (**j**) 1%.

The surface morphology of the fabricated control and TRG membranes is presented in Figure 4. The control membrane has an uneven structure with an irregular distribution of pores. Upon adding TRG to the membranes, the number of pores on the membrane surface increased and became more evenly distributed, resulting in a smoother surface than the control membrane. Figure 4a–d shows that the surface pore size increases as the low concentrations of TRG are added and then decreases at higher concentrations. Therefore, the addition of TRG greatly affects the surface of the fabricated membranes, as it promotes faster de-mixing between the solvent and non-solvent during the phase inversion process, leading to larger pores that are highly interconnected at higher concentrations, as previously reported for sulfonated PES [38]. AFM was employed to study the surface morphology of the prepared TRG membranes through three-dimensional (3D) imaging under tapping mode. A scan size of 10 × 10 µm and 3 × 3 µm was chosen for this analysis. Figure 4e,f shows the 3D images of all the prepared membranes with two different scan sizes. Dark-colored areas in the AFM images correspond to low points like pores and valleys, while bright-colored sites describe high points such as peaks; the right side of the images shows the numbered exact elevation scale, which is consistent with the morphology of PES-GO MMMs [39].

**Figure 5:**
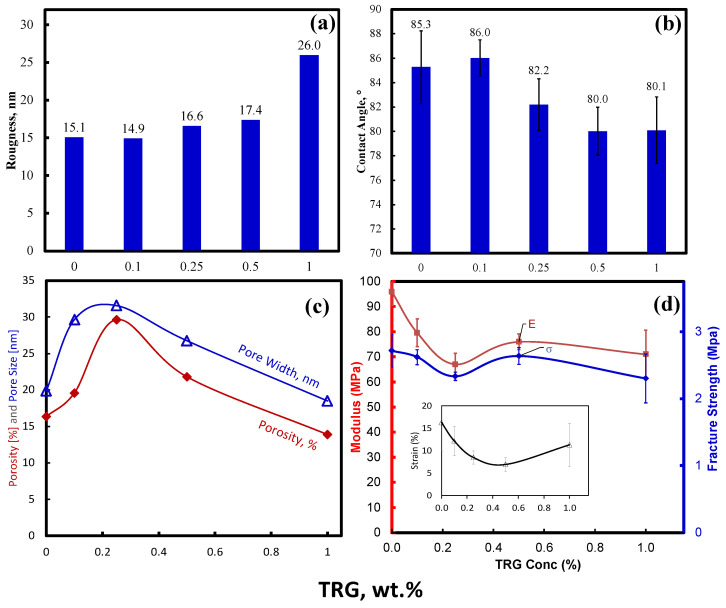
Characterization of PSF-TRG MMMs at different TRG concentrations: (**a**) Surface roughness analysis. (**b**) Contact angle. (**c**) Porosity and pore–width. (**d**) Young’s modulus, fracture strength, and breakage strain.

Figure 5a displays the surface roughness change with different concentrations of TRG membranes. As mentioned earlier, the primary physical parameter obtained from AFM analysis is surface roughness, which describes the deviation from the standard surface to the specified surface; thus, the numerical value of roughness is calculated. A clear trend for the surface roughness is observed, whereby the roughness increases as TRG concentration increases, which is consistent with previously reported data for other fillers [40,41]. The prepared MMMs are more porous than the control membrane; hence, higher roughness, upon adding TRG, is obtained, indicating that the control membrane is the smoothest membrane, with roughness equivalent to 15.1 nm. The 1% TRG membranes showed the highest roughness of 26 nm due to the aggregation of TRG sheets at high loading.

Hydrophilicity is one of the most important characteristics that affect a membrane’s performance. The hydrophilicity of the prepared membranes was investigated by obtaining the average of the multiple contact angle measurements for the same membrane. As is widely known, lower contact angles demonstrate higher hydrophilicity and vice versa. The control membrane exhibited a contact angle of 85.3°, consistent with the literature measurements [42]. The addition of TRG content to PSF control membrane led to a significant reduction in the contact angle, from 85.3° to 80.0° at 0.5 wt.% TRG. This occurred because the additional TRG introduced more hydrophilic groups to the membrane’s surface [43], as illustrated in Figure 5b.

The prepared membranes’ porosity, an essential factor affecting a membrane’s performance, was evaluated using the BET measurement. Figure 5c shows that at low TRG concentrations (0.1 and 0.25 wt.%), there is an increase in membrane porosity and pore size. At higher concentrations (0.5 and 1 wt.%), the porosity and pore size decrease due to the increased viscosity of the PSF polymer solutions, which slows the solvent and non-solvent exchange, leading to reduced pore formation [44]. The 0.25 wt.% achieved the highest porosity and pore size, with an enhancement of porosity of about 81% compared to the control membrane. It is worth noting that the BET method measures porosity in the range of 2 nm to 3 μm, since the membranes contain larger pores. Therefore, the reported porosity is used on a relative basis to compare different membranes.

Good mechanical properties of membranes positively influence the practicality of membranes in large-scale applications [45]. Figure 5d shows the results of Young’s modulus, fracture strength, and strain as a function of TRG concentration. The previous parameters were analyzed using DMA. The control (0 wt.% TRG) membrane exhibited the highest Young’s modulus and fracture strength of 95.9 MPa and 2.7 MPa, respectively. Adding TRG to the PSF matrix decreased Young’s modulus and fracture strength to a minimum of 67 MPa and 2.3 at 0.25 wt.%, resulting in a 30% and 14.8% reduction, respectively. Figure 5d demonstrates the change in the strain, along with an increase in the content of TRG. As can be observed, increasing the concentration of TRG decreases the breakage strain of the membranes significantly, implying the existence of more brittle membranes. The mechanical properties of the fabricated membranes are highly affected by the porosity and pore size distribution. Membranes with low porosity and tiny pores are expected to have superior mechanical properties compared to highly porous membranes. Another factor in determining the mechanical properties of an MMM is the reinforcement ability of the filler due to the high aspect ratio. A tradeoff between the porosity and reinforcement ability of the filler is always expected in establishing the final mechanical properties. The highest Young’s modulus and fracture stress of the control membrane is explained because it exhibited the lowest pore size and porosity among all the MMMs [46]. The incorporation of TRG was predicted to improve the mechanical properties; however, in this study, the porosity effect was more dominant in the mechanical properties than the TRG filler’s reinforcement ability.

In order to study the performance of the membrane in processing oil emulsion, it is important to determine how the TRG loading and emulsion properties affect the performance. Surfactants can affect the stability, droplet size, and charge of the oil emulsion, which, in turn, can influence the permeability, selectivity, and fouling of the membrane [47,48,49]. Therefore, for a concentration of oil in each emulsion set at 100 parts per million (ppm), we varied the TRG loading to observe the impact on the permeability; then, we used surfactant stabilization to study the membrane performance for different emulsion properties.

**Figure 6:**
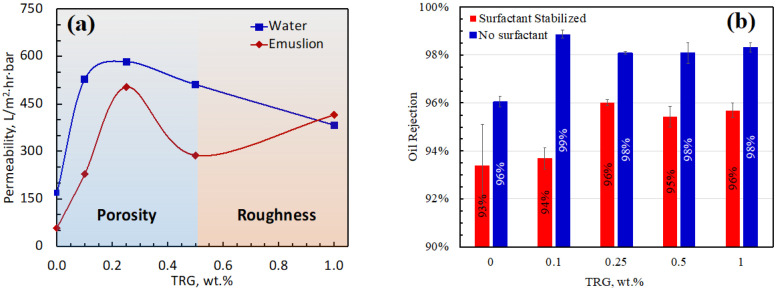
Influence of TRG concentration on permeability and oil rejection of PSF-TRG MMM: (**a**) Water and emulsion permeability analysis: variation in TRG concentration and its impact on water and emulsion permeability in PSF-TRG MMM. (**b**) Oil rejection assessment: effect of TRG concentration on the ability of PSF-TRG MMM to reject oil.

Consequently, the first part of our research is dedicated to setting the concentration of oil in each emulsion at 100 parts per million (ppm) and manipulating the TRG loading. Figure 6a illustrates an interesting phenomenon concerning the impact of TRG addition on the permeability of both water and emulsion. We observe a notable increase in permeability values for both substances, reaching 583 L/m^2^·h bar for water and 504 L/m^2^·h bar for emulsion, with a TRG loading of 0.25 wt.%. This enhanced performance can be attributed to increased porosity, larger pore size, and heightened hydrophilicity, as depicted in Figure 5b,c [50,51]. However, as TRG loading is further increased beyond 0.25 wt.%, a contrasting trend becomes evident. The enhanced loading appears to trigger a decrease in porosity, which subsequently leads to a decrease in permeability for both water and emulsion. This trend is noticeable until a TRG loading of 0.5 wt.%. Intriguingly, beyond a TRG loading of 0.5 wt.%, the permeability trends for water and emulsion diverge. We witness an increase in the permeability of the emulsion, even as water permeability continues to decline. This phenomenon could possibly be attributed to a further reduction in porosity accompanied by an increase in surface roughness. The heightened surface roughness potentially enhances sorption capabilities, which, in turn, boosts emulsion permeability. However, the reduction in porosity appears to exert a negative influence on the permeability of both water and emulsion. The complex interplay of these factors sheds light on the nuanced impact of TRG loading on permeability under different conditions.

We used a surfactant, Sodium dodecyl sulfate (SDS), to alter the oil emulsion’s properties, thereby enabling a comprehensive study of the membrane’s performance under emulsion properties. It is noteworthy that each TRG MMM demonstrated improved oil rejection compared to the control membrane, as shown in Figure 6b. With the application of surfactants, specifically, SDS, the oil rejection was found to be 93.4%. Without the use of SDS, oil rejection increased slightly to 96%. Moreover, it is worth mentioning that the oil concentration in the permeate of every TRG membrane fabricated was less than that in the control membrane. A remarkable oil rejection peak of 98.9% was observed with 0.1 wt.% TRG loading. This peak performance with 0.1 wt.% TRG loading may be primarily attributed to the membrane’s porosity [52], as hydrophilicity and roughness appeared to have an insignificant impact at this specific loading level. Interestingly, oil rejection levels seemed to decline as TRG loading increased beyond this point, despite the corresponding rise in porosity. A plausible explanation for this phenomenon could be the disproportionate increase in pore size relative to the emulsion droplets [53,54]. This could, in turn, have facilitated the improved passage of oil, leading to a reduction in oil rejection.

### 3.3. Effect of TRG Concentration on the Permeance and Selectivity of Air-Dehumidification Membrane

The inclusion of 0.25 wt.% TRG leads to an enhanced humidity removal by 2.4% as shown in Table 1. A more detailed analysis of the effects of TRG on the membrane’s properties provides insights into the reasons behind this enhancement. Analysis of the surface morphology (Figure 2) reveals that incorporating TRG created a more uniform distribution of pores and increased pore sizes at lower TRG concentrations. This is associated with the enhanced porosity seen at 0.25 wt.% TRG, a value approximately 81% higher than that of the control PSF 15 wt.% sample.

The rise in hydrophilicity can also be correlated with the TRG concentration. The second section reveals that hydrophilicity increases as TRG content increases, evidenced by a decrease in the contact angle. This aligns with the observed affinity of water vapor molecules for the 0.25 wt.% TRG membrane’s surface. This improved hydrophilicity and porosity enhance WVP, which sees an increase of 2900 GPUs for WVP from 13,710 GPU as shown in Table 1. Interestingly, while there is a substantial increase in porosity and hydrophilicity, the TRG’s effect on selectivity remains negligible. The second section suggests this could be due to the larger, interconnected pores formed at low TRG concentrations, which may not significantly discriminate between different molecules.

## 4. Conclusions

We successfully synthesized TRG through thermal exfoliation and incorporated it into a PSF matrix to fabricate MMMs using the phase inversion method. The synthesized membranes were characterized and tested using various techniques, including contact angle measurements, dynamic mechanical analysis, SEM, AFM, pure water fluxes, and oil separation analysis. The introduction of TRG into the PSF matrix resulted in modified membranes with a more porous structure than the control membrane, as evidenced by porosity measurements and SEM analysis. The hydrophilicity of the MMMs was significantly improved, as indicated by the lower contact angle measurements. It was observed that the mechanical properties of the modified membranes, such as Young’s modulus and fracture strength, decreased due to the increased porosity, which played a more significant role in determining the mechanical properties than the reinforcement ability of TRG. In terms of performance, the MMM with 0.25 wt.% TRG exhibited the highest permeability enhancement of 71% compared to the other MMMs. However, it was noted that, at higher TRG loadings, agglomeration occurred, leading to a significant decrease in permeability. All TRG MMMs demonstrated higher oil rejection rates than the control membrane, with the highest oil rejection achieved by the 0.1 wt.% TRG MMM at 98.9%.

The prepared TRG/PSF UF MMMs showed improved water fluxes and separation efficiencies, making them suitable for applications in oily wastewater treatment. Additionally, the incorporation of TRG significantly increased the permeance of the membranes for air-dehumidification, reaching a permeance of 16,500 GPU, making them highly permeable membranes in this regard. Overall, this study highlights the potential of using TRG as a promising additive in MMMs, offering enhanced performance characteristics for various applications.

To distill the key contributions of this work, the following points effectively summarize our research:

- Synthesized TRG via a simple thermal exfoliation method and successfully incorporated it into PSF to fabricate an MMM.
- Investigated the effect of TRG loading on membrane morphology, hydrophilicity, porosity, roughness, and mechanical properties.
- Demonstrated that adding TRG led to increased membrane porosity and hydrophilicity compared to the control PSF membrane.
- Showed that TRG incorporation improved water flux by up to 70% and oil rejection by up to 98.9% for the PSF-TRG MMMs compared to the control PSF membrane.
- PSF-TRG MMM with 0.25 wt.% TRG exhibited the optimal combination of enhanced permeability (70% higher) and selectivity (higher oil rejection) for oil/water separation.
- PSF-TRG MMMs demonstrated over 20% increased WVP compared to the control PSF membrane, indicating promise as air-dehumidification membrane supports.
- Provided a simple and effective method to synthesize and incorporate TRG to engineer high-performance polymeric MMMs suitable for oil/water separation and air-dehumidification applications.

## Figures and Tables

**Table 1:** Water vapor transmission results for PSF and 0.25 wt.% TRG.

Membrane Type	Absolute Humidity (kg/m^3^)		Humidity Removed	Water Vapor Permeance (GPU)		H_2_O/Air Selectivity
	Feed	Retentate	%	Water	Air	
PSF (Control)	0.0271	0.0191	29.4	13,710 ± 233	2010 ± 130	7
PSF 0.25% TRG	0.0255	0.0174	31.8	16,650 ± 773	3520 ± 165	5

## Data Availability

Data will be available upon a reasonable request to the corresponding author.
